# A unique horizontal gene transfer event has provided the octocoral mitochondrial genome with an active mismatch repair gene that has potential for an unusual self-contained function

**DOI:** 10.1186/1471-2148-11-228

**Published:** 2011-07-29

**Authors:** Jaret P Bilewitch, Sandie M Degnan

**Affiliations:** 1 School of Biological Sciences, University of Queensland, St. Lucia, Brisbane, Queensland, Australia

## Abstract

**Background:**

The mitochondrial genome of the Octocorallia has several characteristics atypical for metazoans, including a novel gene suggested to function in DNA repair. This *mtMutS *gene is favored for octocoral molecular systematics, due to its high information content. Several hypotheses concerning the origins of *mtMutS *have been proposed, and remain equivocal, although current weight of support is for a horizontal gene transfer from either an epsilonproteobacterium or a large DNA virus. Here we present new and compelling evidence on the evolutionary origin of *mtMutS*, and provide the very first data on its activity, functional capacity and stability within the octocoral mitochondrial genome.

**Results:**

The *mtMutS *gene has the expected conserved amino acids, protein domains and predicted tertiary protein structure. Phylogenetic analysis indicates that *mtMutS *is not a member of the *MSH *family and therefore not of eukaryotic origin. *MtMutS *clusters closely with representatives of the *MutS7 *lineage; further support for this relationship derives from the sharing of a C-terminal endonuclease domain that confers a self-contained mismatch repair function. Gene expression analyses confirm that *mtMutS *is actively transcribed in octocorals. Rates of mitochondrial gene evolution in *mtMutS*-containing octocorals are lower than in their hexacoral sister-group, which lacks the gene, although paradoxically the *mtMutS *gene itself has higher rates of mutation than other octocoral mitochondrial genes.

**Conclusions:**

The octocoral *mtMutS *gene is active and codes for a protein with all the necessary components for DNA mismatch repair. A lower rate of mitochondrial evolution, and the presence of a nicking endonuclease domain, both indirectly support a theory of self-sufficient DNA mismatch repair within the octocoral mitochondrion. The ancestral affinity of *mtMutS *to non-eukaryotic *MutS7 *provides compelling support for an origin by horizontal gene transfer. The immediate vector of transmission into octocorals can be attributed to either an epsilonproteobacterium in an endosymbiotic association or to a viral infection, although DNA viruses are not currently known to infect both bacteria and eukaryotes, nor mitochondria in particular. In consolidating the first known case of HGT into an animal mitochondrial genome, these findings suggest the need for reconsideration of the means by which metazoan mitochondrial genomes evolve.

## Background

Animal mitochondrial genomes are generally conserved in structure, approximate size and gene content. The circular genome of the so-called higher metazoans typically encodes 13 proteins, 22 transfer RNAs and two ribosomal RNAs, while lacking introns [1,2]. Variation in genome size usually derives only from length variation in the single large non-coding region, so that most mitochondrial genomes range in size from 14 to 17 kb [1,2]. The phylum Cnidaria, however, provides many interesting and diverse exceptions to this pattern. For example, medusozoans (jellyfish and hydroids; Classes Cubozoa, Scyphozoa, Staurozoa and Hydrozoa) are unique among animals in having a linearized mitochondrial DNA (mtDNA) [3,4]. Hexacorals (Anthozoa: Hexacorallia) are unique in that their mitochondrial *COI *and *ND5 *genes contain introns, and anthozoan mitochondrial genomes on the whole are known to contain a greatly reduced complement of transfer RNA genes (only 1 or 2 tRNAs) [5-9]. Among the octocorals (Anthozoa: Octocorallia), the mitochondrial genome is known to have undergone inversion and reorganization at least three times [10,11], including twice within a single family [11]. These changes in octocoral gene order appear to have resulted from intramitochondrial recombination [10], which itself was believed to be unlikely in animals until its discovery in 1997 [12]. As more mitochondrial genome data become available for cnidarians, and for the Octocorallia in particular, it seems that fewer generalizations of mitochondrial structure and evolution seem applicable to these basal metazoans.

Indeed, when the first complete octocoral mitochondrial genomes were sequenced in 1995, another surprise was revealed. The mtDNA of *Renilla kolikeri *and *Sarcophyton glaucum *(Anthozoa: Octocorallia) [5,13] was observed to contain the standard anthozoan complement of oxidative metabolic genes - 13 protein-coding genes, two rRNAs and a single tRNA - but both genomes also contained an unexpected open reading frame (ORF) [5,14,15]. Almost 16% of the mtDNA (2949/18453 bp) was occupied by a novel ORF that to this day has not been found in any mitochondrial genome outside of the Octocorallia [5]. Based on 19.7% similarity of the translated sequence to the yeast nuclear Mutation Suppressor Homolog 1 (*MSH1*) gene, it was suggested that the novel ORF might be active in DNA mismatch repair (MMR) and thus it was first termed '*mtMSH*' as a mitochondrial homolog of the eukaryotic *MSH *family [14]. Alternative names have since been used for this octocoral gene to infer relationship specifically to the *MSH1 *family ("*msh1*" [16,17]) or to encompass broad taxonomic ranges ("*mtMutS*" [18]), including MMR genes from both prokaryotes (*MutS*) and eukaryotes (*MSH*). We hereafter preferentially use the name '*mtMutS' *to refer to the octocoral mitochondrial ORF, since it makes the fewest *a priori *assumptions of homology to either eukaryotic *MSH *or prokaryotic *MutS *MMR gene families, specifically.

Paradoxically, while *MutS *and *MSH *function in reducing error rates in DNA replication in eubacteria and eukaryotes, respectively [19], *mtMutS *itself possesses the highest level of systematically-informative variation amongst any protein-coding mitochondrial gene examined to date within octocorals [20]. As such, it has played a central role in molecular systematic efforts to revise the taxonomic classification of the Octocorallia. Its utility in phylogenetics was first demonstrated in a species-level analysis [16] and has subsequently been extended to more comprehensive studies of octocoral genera [17] and subfamilies [21]. Revisionary systematics and the classification of newly described species have also used sequences of *mtMutS*, either alone [22] or in combination with nuclear [23,24] or other mitochondrial [25,26] sequences. Despite being rapidly embraced as a phylogenetic tool, however, *mtMutS *is the only animal mitochondrial gene for which both functionality and origin remain equivocal.

**Figure 1:**
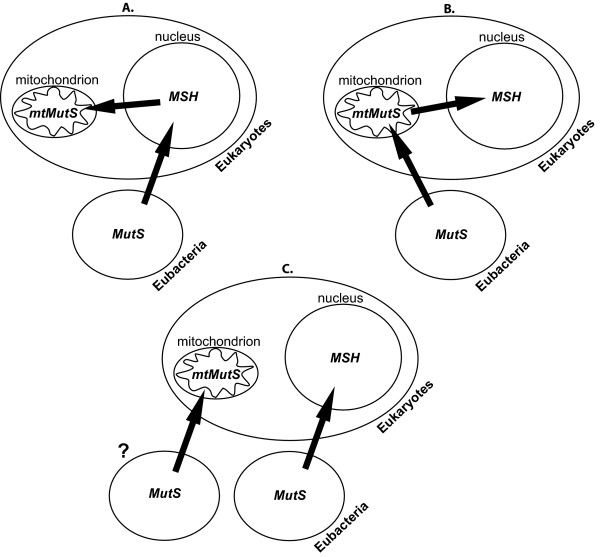
**Hypotheses of *mtMutS *origins**. Three previously-proposed hypotheses concerning the proximate origins of octocoral *mtMutS*. (A) *mtMutS *originated as a nuclear *MSH *gene and was subsequently transferred to the mitochondrial genome in octocorals [15]; (B) *mtMutS *entered the ancestral eukaryotic cell via endosymbiosis of the mitochondrion-like bacterial ancestor, and was subsequently transferred to the nucleus as *MSH *and lost from the mitochondrion of all other eukaryotes [18]; (C) Although both involved a bacterial *MutS *precursor, *mtMutS *was acquired by the ancestral octocoral mitochondrion independent of the means by which eukaryotic nuclei acquired *MSH *[32].

The observation that the *mtMutS *gene is present in all octocorals [17], yet absent from all examined members of the hexacoral sister clade [7,8] has been instrumental in the formulation of numerous theories regarding its origin (Figure 1). Since all known eukaryotic *MSH *genes are found in the nucleus, it was first postulated that the presence of an *MSH*-like gene in the octocoral mitochondrial genome most likely resulted from gene transfer from the octocoral nucleus (Figure 1A) [15]; this event would have occurred in the common octocorallian ancestor after its divergence from hexacorals. It was further hypothesized that, as a homolog of the *MSH1 *gene [14], *mtMutS *would likely play a role in stabilizing mtDNA [27,28]. This latter hypothesis is indirectly supported by observations of low levels of DNA sequence variation among other mitochondrial genes commonly used for octocoral population genetics and barcoding efforts [20]. However, although gene transfer from the mitochondrion to the nucleus is well-documented, transfer in the opposite direction, such as that initially proposed for *mtMutS *[15], has never been confirmed [29]. Different interpretations of the relationship of *mtMutS *to *MSH1 *suggested that the eukaryotic *MSH *family originated from a nuclear *MSH1 *ancestor, which was itself transferred from the genome of the endosymbiont precursor of the mitochondrion (Figure 1B) [18]. Under this scenario, the presence of *mtMutS *in the octocoral mitochondrion represents a relict intermediate between the cellular internalization of bacterial *MutS *and nuclear eukaryotic *MSH*. However, with more comprehensive taxonomic sampling, the phylogenetic position of *mtMutS *relative to *MSH1 *changes, further diminishing the likelihood it represents a nuclear-mitochondrial transfer event in either direction. A broad-scale study including representative sequences of six eukaryotic *MSH *families and two bacterial *MutS *lineages placed *mtMutS *not as a close relative of *MSH *genes, but instead at the base of the *MutS2 *family, which contains prokaryotic and plant *MutS *genes with unknown functions [30,31].

A more recent analysis proposed the closest relative of the octocoral *mtMutS *to be the *MutS *sequence from the *Acanthamoeba polyphaga *nucleocytoplasmic large DNA virus (NCLDV or 'girus'), closely followed by *MutS *sequences from epsilonproteobacteria [32]. Although no other eukaryotic sequences were included in the analysis, the high level of identity between *MutS *from the NCLDV and *mtMutS *(25%), and the presence of a C-terminal HNH endonuclease domain in both, were taken as evidence of common ancestry. This raised the hypothesis that octocorals might have acquired *mtMutS *via horizontal gene transfer (HGT) from a NCLDV ancestor into the common ancestor of Octocorallia (Figure 1C). An HGT origin of *mtMutS *is further supported by the most recent and most comprehensive analysis of both *MSH *and *MutS *evolution, which included five more NCLDV sequences [33]. This study provided very strong support for the lineage containing octocoral, epsilonproteobacterial and viral *MutS *sequences, but was unable to discriminate between the bacterial or the viral lineage as the sister group to *mtMutS *[33]. Thus the most recent common ancestor to *mtMutS *remains unknown, although it appears almost certain to be either viral or bacterial - not eukaryotic - in origin.

Here we provide further tests of the HGT hypothesis of origin by examining the homology, diversification and evolutionary patterns of the *mtMutS *gene from multiple octocorals. First, we reconstruct *MutS *phylogenies using all conserved sections of the gene, rather than only the single domain used previously [33], thus providing more robust support for the HGT origin. Horizontal gene transfer of a *mtMutS *precursor from a non-metazoan donor in turn raises multiple questions pertaining to the acquisition, function and evolution of the octocoral gene. In particular, evolutionary theory predicts that the successful integration of HGT- acquired genes into a host genome requires both gene activity and gene longevity [34]. Second, in addressing these predictions, we ask whether the octocoral *mtMutS *gene is active, and how it has diversified over evolutionary timescales within the foreign host genome [35]. In combination, these analyses significantly expand our understanding of a novel gene that has persisted within octocorals since their most recent common ancestor.

## Results

### A HGT origin of *mtMutS *in the common ancestor of Octocorallia

PSI-BLAST returned a set of epsilonproteobacteria and nucleocytoplasmic large double-stranded DNA virus (NCLDV) *MutS *hits as best matches to complete, translated octocoral *mtMutS *sequences present in GenBank.

**Figure 2:**
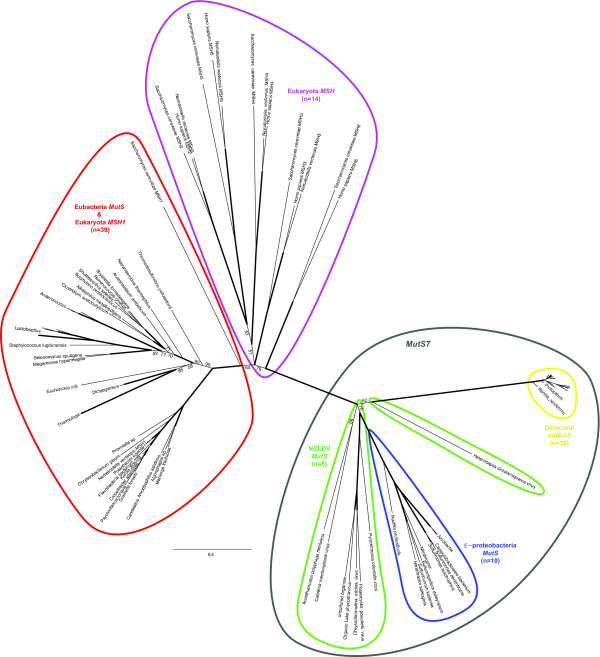
**Bayesian phylogenetic tree of mtMutS, MutS and MSH gene families**. The majority-rule consensus unrooted tree of four runs of 10^7 ^MCMC generations with samples drawn every 1000 generations and 3^6 ^generations discarded as burn-in. Branches in bold had >95% support while other branches are labelled with lower posterior probabilities. Maximum parsimony analysis produced an unrooted tree with similar general topology (not shown). Coloured ovals delineate the major taxonomic groups or gene families. Taxon sample sizes are given in parentheses for each circled clade.

Both Bayesian (Figure 2) and maximum parsimony (not shown) phylogenetic analysis of an alignment of *mtMutS *with *MutS *and eukaryotic *MSH *representatives (Additional file 1) produced an unrooted tree with strong support for the major *MSH *and *MutS *lineages. All octocoral *mtMutS *sequences were monophyletic (100% posterior probability support; Figure 2, yellow oval), with a basal node connected to the remainder of the tree by a long uninterrupted branch. One of the paraphyletic NCLDV *MutS *sequences, for the *Heterocapsa circularisquama *virus, (Figure 2, green oval) formed the sister group to octocorals, although posterior probability support for this placement was a low 62%. The next most probable alternative arrangements of both the *Acanthamoeba polyphaga *virus and *Cafeteria roenbergensis *virus or just the *A. polyphaga *virus *MutS *lineages as sister to *mtMutS *received 20.6% and 12.6% support, respectively. *MtMutS *as sister to a clade of all NCLDV viruses and epsilonproteobacteria, or to a clade of just the epsilonproteobacteria, received less than 5% support (data not shown). A clade of octocoral *mtMutS*, epsilonproteobacteria *MutS *and viral *MutS *received 100% support, indicating the exact sister group of octocorals remains equivocal but contains viral and possibly bacterial lineages with high confidence.

All eukaryotic sequences, with the exception of *MSH1*, formed gene family-specific monophyletic *MSH *clades with over 95% support (Figure 2, purple oval). Of all the *MSH *families, *MSH6 *shared the most recent common ancestor with octocoral *mtMutS *in our Bayesian phylogeny. However, none of the eukaryotic *MSH *families formed a monophyletic lineage with octocorals and, with the exception of *MSH2*-*MSH4*-*MSH5*, formed polyphyletic inter-familial relationships amongst themselves. Additionally, the two included sequences of eukaryotic *MSH1 *(one each from *Nematostella *and yeast) formed polyphyletic associations amongst the eubacterial *MutS *sequences at the distal end of the Bayesian tree to octocoral *mtMutS *(Figure 2, red oval), indicating they are highly divergent. These arrangements preclude hypotheses of an immediate common ancestry between *mtMutS *and *MSH1 *(Figure 1A,B.)

A lack of monophyly between *mtMutS *and any *MSH *family, combined with the phylogenetic arrangement of *mtMutS *within the *MutS7 *lineage (Figure 2, grey oval) provides further support to a hypothesis of non-eukaryotic origin of *mtMutS *in the most recent common ancestor of extant octocorals. The sister organism(s) from which the *mtMutS *precursor was obtained was not identified with confidence in the phylogeny, but the placement of *mtMutS *within *MutS7 *strongly supports a hypothesis of horizontal gene transfer.

### *MtMutS *predicts a functional protein structure

**Figure 3:**
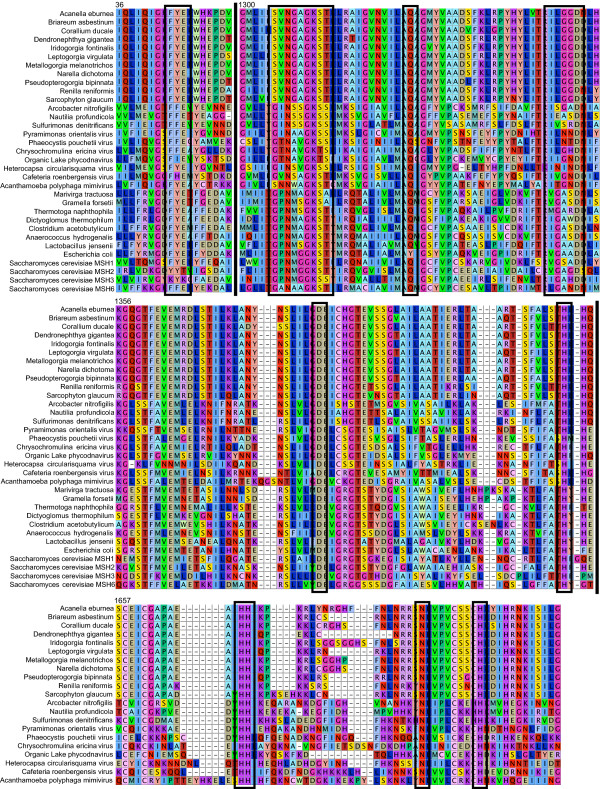
**Conservation of active residues among mtMutS, MutS and MSH amino acid sequences**. An amino acid alignment of a reduced set of taxa included in the phylogenetic analysis, illustrating conserved positions or motifs (black boxes) known to function in MutS/MSH-mediated MMR or HNH endonuclease activity. Numbers above alignment blocks indicate the position within the alignment, relative to the 5'-end of octocoral sequences. Vertical lines separate the three regions of homology, in order: Domain I, Domain V and the HNH endonuclease domain. Taxa lacking the HNH domain were omitted from the latter alignment.

Comparisons of translated octocoral *mtMutS *sequences with bacterial *MutS *and eukaryotic *MSH *genes identified conserved residues consistent with MutS-protein functional sites in all octocoral sequences (Figure 3). An invariant phenylalanine is located at position 44, consistent with a MutS Domain I DNA mismatch detection site [36,37]. Positions 1305 to 1313 contain a SVNGAGKST motif consistent with a Walker A loop of a MutS ATPase domain (Domain V) [36-38]. Walker B- (hhhhD/E; positions 1348-1352), Q- (glutamine; position 1326), H- (histidine; position 1425) and D-loop (asparagine; position 1387) motifs of a MutS Domain V are also invariant among all octocoral sequences.

**Figure 4:**
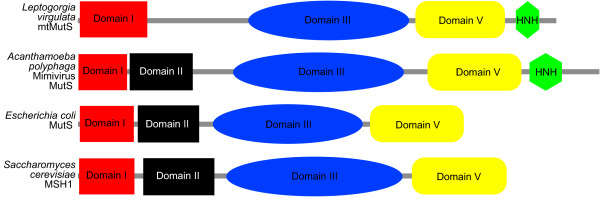
**A comparison of predicted protein family domains in mtMutS, MutS and MSH amino acid sequences**. The results of SMART database queries for an exemplar octocoral *mtMutS *sequence (GenBank Acc. # AAP92775) *vs. *the predicted domain organization of the *Acanthamoeba polyphaga *mimivirus (YP_142713), *E. coli *(CAQ33065) and yeast (NP_011988). 'Domain I', 'II', 'III' and 'V' refer to *MutS/MSH*-specific domains; 'HNH' refers to the HNH endonuclease domain.

All 30 octocoral *mtMutS *sequences included in our analysis contain three of the expected 'pfam' domains: a MutS Domain III ('MUTSd'), a MutS ATPase Domain V ('MUTSac') and an HNH endonuclease domain ('HNHc') (Figure 4). A fourth MutS Domain I ('MutS_I') is found in all but three sequences (data not shown). However, a homologous amino acid sequence for Domain I is apparent in all 30 octocoral sequences (Figure 3). There is no evidence of domain shuffling, with all sequences showing a consistent order of Domain I - Domain III - Domain V - HNH endonuclease. We found no evidence of Domains II and IV in any octocoral *mtMutS *sequence and their presence is likely to be unnecessary for protein activity since they are absent from nearly all *MutS *families aside from *MutS1 *[[33]: Figure 2].

As previously reported for *S. glaucum *[31], the HNH endonuclease domain detected by SMART at the C-terminal end of all octocoral *mtMutS *sequences contains characteristic histidine-histidine-asparagine-histidine (HHNH) amino acids at positions 1676, 1677, 1709 and 1718, respectively (Figure 3). These residues are conserved with HNH-containing *MutS *sequences found among the epsilon-proteobacteria and NCLDV sequences. HNH domains were not detected by SMART in any other queried MSH or MutS protein sequences, providing further evidence of common ancestry of *mtMutS *and other *MutS7 *genes.

**Figure 5:**
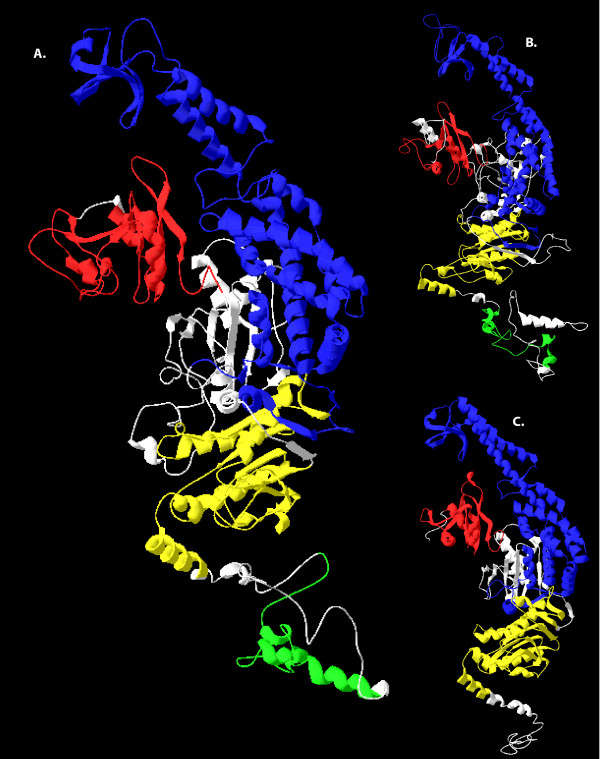
**The predicted tertiary protein structure of mtMutS and its similarity to predicted and experimentally determined MutS structures**. (A) The predicted monomer structure of the mtMutS protein, as determined by the I-TASSER database. (B) The predicted monomer structure of the HNH-containing *Acanthamoeba polyphaga *mimiviral MutS protein. (C) The crystal structure of a monomer of MutS from *E. coli*, as determined by [36]. Colours correspond to the domains identified in Figure 4: Red = Domain I, Blue = Domain III, Yellow = Domain V, Green = HNH endonuclease domain.

Protein tertiary structures predicted from all octocoral *mtMutS *sequences are also consistent with the structure of the known, functional MutS monomer in *E. coli *(Figure 5), although the presence of a C-terminal HNH endonuclease domain is more similar to the tertiary structure and domain organization we predicted for *A. polyphaga *mimivirus MutS (Figure 5B). The predicted relative positions of Domain I and Domain III in octocoral sequences support their roles as a mismatch-specific central clamp and a non-specific upper DNA clamp, respectively (see also [36]). Domain V forms the structural core of mtMutS, with the HNH endonuclease as a C-terminal 'tail'. To our knowledge, these results represent the first report of predictive reconstruction and comparison of mtMutS protein structure in octocorals and support a functional role of *mtMutS *in DNA mismatch repair.

### *MtMuts *is expressed and available for translation

**Figure 6:**
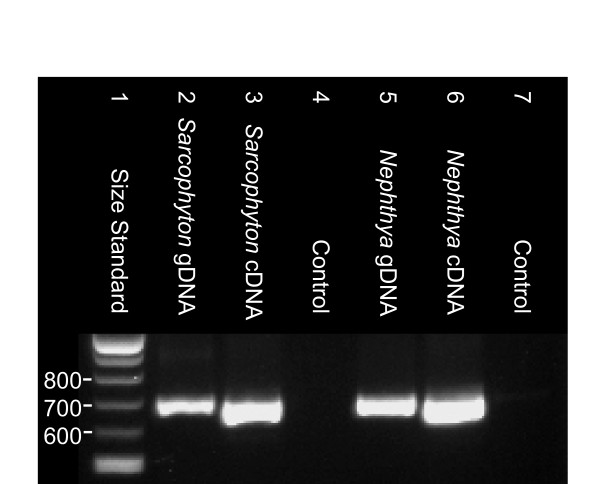
**Gel electrophoresis results of PCR amplification of *mtMutS *from genomic DNA and transcribed mRNA**. A 1.5% TBE gel displaying the results of PCR amplification of the 5'-end of the *mtMutS *gene from genomic mtDNA (lanes 2 & 5) and reverse-transcribed mRNA (lanes 3 & 6). Controls (lanes 4 & 7) of corresponding mRNA samples lacking polymerase in reverse-transcription reactions were included to demonstrate that cDNA PCR products were not of genomic DNA origin.

A 687 bp DNA product was produced by amplification of *mtMutS *from both gDNA and cDNA using PCR primers specific to the 5'-end of the gene (Domain I) (Figure 6). Controls for both genomic DNA contamination of RNA (cDNA controls lacking reverse transcriptase) and for foreign DNA contamination (no template added) failed to amplify, confirming that cDNA PCR products were of mRNA origin. The sequences of cDNA and gDNA PCR products from *Isis hippuris*, *Nephthya *sp. and *Sarcophyton ehrenbergi *were all confirmed by BLAST as mitochondrial *mtMutS *sequences. No post-transcriptional modification was detected in comparisons of cDNA and gDNA sequences. These results provide the first evidence that *mtMutS *is transcribed in a variety of octocorals and that the mRNA is available for translation into a potentially functional protein.

### *MtMutS *has evidence of higher rates of mutation than other octocoral mt genes

Alignments of 1597 bp in 169 taxa, 3967 bp in 410 taxa and 3426 bp in 509 taxa were generated for octocoral *COI*, hexacoral *COI *and octocoral *mtMutS *mtDNA sequences, respectively, using all available data present in GenBank. Alignments of 1737 bp in 224 taxa and 1871 bp in 145 taxa were generated for hexacoral and octocoral 18S nuclear DNA sequences, respectively. Mann-Whitney *U*-tests indicated highly-significant differences (p < 0.0001) between the means of pairwise genetic differences in mtDNA sequence comparisons of octocoral *mtMutS vs. *octocoral *COI *and octocoral *COI vs. *hexacoral *COI *and between nuclear DNA sequence comparisons of octocoral 18S *vs. *hexacoral 18S. Tests using only taxa with less than 10% missing data in each comparison yielded identical significant results (data not shown), thus the inclusion of taxa with incomplete gene sequences did not alter the results of the full comparisons.

**Figure 7:**
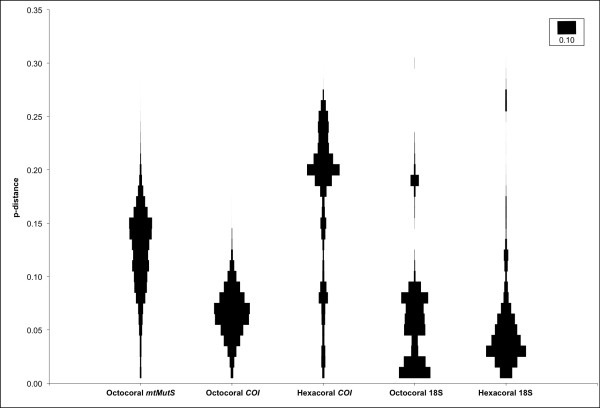
**Relative frequencies of pairwise genetic distances among octocoral and hexacoral mitochondrial and nuclear DNA sequences**. A comparison of the distributions of uncorrected pairwise distances of DNA alignments of 509 sequences of octocoral *mtMutS*, 169 sequences of octocoral *COI*, 410 sequences of hexacoral *COI*, 224 sequences of hexacoral 18S and 145 sequences of octocoral 18S. Widths of each row correspond to the relative frequency of each category of pairwise distance. The scale bar (inset) indicates a relative frequency of 10%.

The distances among the octocoral *COI *sequences display both the smallest range and modal value among mitochondrial datasets, whereas the hexacoral *COI *sequences display the largest range and the highest modal value (Figure 7). The octocoral *mtMutS *sequences display both an intermediate range and mode between octocoral *COI *and hexacoral *COI*. The hexacoral *COI *distribution is negatively skewed (Figure 7), with greater distances attributed to inter-ordinal comparisons and, within the Scleractinia, distances between the 'complex' and 'robust' clades [39] (data not shown).

Among the nuclear 18S sequence comparisons, octocorals display a significantly higher mean pairwise distance than hexacorals (p < 0.0001), opposite to the pattern observed for the mitochondrial genome. Both octocoral and hexacoral 18S distributions were positively skewed and displayed, respectively, the highest and second-highest distances out of all examined datasets. However, both 18S datasets had modal values and the majority of their distributions in a range of distances similar to the distribution of octocoral *COI *(Figure 7).

These patterns indicate an overall reduced rate of evolution of octocoral *COI *relative to hexacoral *COI *since the time that they diverged from their last common ancestor. Given the corroboration of previous observations of low divergence levels in other mitochondrial genes [20,40,41], we may extrapolate our observation to the mitochondrial genome as a whole. However, the *mtMutS *gene itself displays significantly higher pairwise distances than *COI*, although its modal divergence level is still low compared to that observed between inter-ordinal taxa of scleractinians (Figure 7). Furthermore, the results of the 18S comparison indicate that the reduction in rates of octocoral mitochondrial mutation may not extend to the nuclear genome. Octocorals may, conversely, display higher rates of nuclear mutation than hexacorals, as displayed for our 18S comparison (Figure 7).

## Discussion

### Non-eukaryotic origins of *mtMutS *and mitochondrial HGT

The mitochondrial targeting of MSH1 plus initial observations of sequence similarity between *MSH1 *and *mtMutS *formed the basis of early conclusions that the two genes were orthologous [15]. However, this phylogenetic association appears to be artifactual, due to insufficient sequence sampling from prokaryotic sources in these initial studies. The continued expansion of available *MutS *sequences from an array of prokaryotes allowed a new phylogenetic arrangement for *mtMutS *- one that placed it in an association with the epsilonproteobacteria and giant double-stranded nucleo-cytoplasmic viruses (NCLDV's or "giruses") [32] in one of four new *MutS *lineages, namely *MutS7 *[33]. Members of the *MutS7 *lineage are united by Domains I, III and V of MutS plus a C-terminal HNH endonuclease domain. In contrast, *MSH1 *and other members of its *MutS1 *lineage contain *MutS/MSH *domains II and IV and lack an endonuclease domain [33]. Our phylogenetic analysis provides further support for this placement of octocoral *mtMutS *within *MutS7 *with high levels of confidence (Figure 2), and provides conclusive evidence that the octocoral *mtMutS *gene does not share an immediate common ancestor with any eukaryotic *MSH *family, including *MSH1*. The exclusion of the HNH domain from our phylogenetic analysis indicates the tree topology is based upon evolution of homologous *MutS *domains and not on this *MutS7 *synapomorphy. If *mtMutS *is active in mitochondrial MMR, this similarity in function with *MSH1 *would likely be the result of convergent evolution between paralogous genes. For these reasons, the octocoral mitochondrial *MutS *gene should no longer be referred to as '*msh1*', as in the current systematic literature (e.g. [20,25,41]), since it carries the connotation of eukaryotic origins. We recommend the adoption of the name *mtMutS*, after studies which first identified it as a unique mitochondrial MutS gene (this study; [30,31]). Even more specifically, *mtMutS7 *could be used to further identify it as a member of the *MutS7 *HNH endonuclease-containing family [33].

The evidence for a non-eukaryotic origin of *mtMutS *in octocorals rules out the possibility of either a mitochondrion-to-nucleus [18] or nucleus-to-mitochondrion [15] translocation hypothesis in its relationship to *MSH *families (see Figure 1B,C). This also necessitates a reinterpretation of the origins of *MSH *in eukaryotes. It has been proposed that the endosymbiotic incorporation of a mitochondrion-like prokaryotic cell brought *MutS *into the primordial eukaryotic cell (as *MSH1*), with translocation to the nucleus and a series of gene duplications producing the remaining *MSH *families [18]. While *MSH1 *was proposed to be a basal family among all *MSH*s, evidence for it once existing within the mitochondrial genome was based on apparently artifactual phylogenetic associations with *mtMutS*. In addition to our findings (Figure 2), more comprehensive *MutS/MSH *phylogenies [30,33] also indicate that *MSH1/MutS1 *is a highly-derived lineage relative to the other eight families of prokaryotic *MutS *and six families of eukaryotic *MSH*. Thus, although a prokaryotic origin of *MSH *is supported by all current phylogenetic hypotheses [18,30,33], evidence for an intermediary role of the mitochondrion in this process is lacking.

### Determining the vector of *mtMutS *HGT

The affinity of a monophyletic octocoral *mtMutS *lineage to the *MutS7 *family containing *MutS *from epsilonproteobacteria and NCLDVs supports the hypothesis of horizontal gene transfer (HGT), as initially suggested [18] and later evidenced [32,33]. The presence of the HNH endonuclease domain supports a common ancestry of *MutS *in these three disparate groups of organisms [32,33], but the direction and means by which the gene was distributed remain questionable. The *MutS7 *lineage could have origins either within the basal Eukaryota or the Eubacteria, given that it occupies a clade in-between the *MutS1/MSH *lineages and the other seven *MutS *families [[33]: Figure 1]. Within the *MutS7 *lineage, the branching order of the octocorallian, bacterial and viral *MutS *lineages can provide clues into the origins of the gene family. Unfortunately, neither the previous comprehensive phylogenetic analysis [33] nor our current study produced phylogenies with strongly supported topologies for the branching order of *MutS *from Octocorallia, NCLDVs and epsilonproteobacteria. The octocoral *mtMutS *and epsilonproteobacterial *MutS *lineages are both monophyletic with high posterior probability, as is the entire *MutS7 *lineage, but whether the NCLDV, the epsilonproteobacteria or both are the sister group to octocorallian *mtMutS *can not yet be determined (Figure 2; [[33]:Figures 1a, S2]). Although less taxonomically-comprehensive for octocorals, a previous study [[32]: Figure 5] provided 89% bootstrap support for *A. polyphaga *mimiviral *MutS *as the sister lineage to octocoral *mtMutS*, with the epsilonproteobacteria forming a basal *MutS7 *divergence. On this finding, they proposed a scenario whereby *mtMutS *was transferred from a microbial host into the mitochondrion of the common octocorallian ancestor via a NCLDV vector.

Although viruses infecting the mitochondrial genome ('mitoviruses') have been documented, they have so far been limited to single- or double-stranded RNA viruses and are so far only known to infect fungi [42]. Double-stranded DNA viruses, such as the NCLDVs, are not known to infect host mitochondria, thus a mechanism by which a viral *MutS *could have entered an octocoral mitochondrion is currently lacking. Interestingly, mitochondria are known to migrate to viral assembly sites in infected primate cells [43,44]. If similar responses occurred in NCLDV-infected cells, it would place the host mitochondrial genome in proximity to viral DNA. At this time, however, it is unknown whether any *MutS7*-containing NCLDV is capable of infecting an octocorallian cell. Furthermore, although NCLDVs infect a wide range of hosts [33], none of them are bacterial and no examples exist of any virus that directly infects both prokaryotic and eukaryotic cells.

An alternative means for octocorals acquiring *mtMutS *through HGT may not involve NCLDVs at all. Diverse arrays of bacterial genotypes are known to associate with the surfaces and tissues of marine invertebrates, including anthozoans [45,46]. These microbial communities can include endosymbionts [47,48], which may confer advantages to host cells such as the fixation of nitrogen [48]. HGT between bacteria is thought to be common in the marine environment [49] and has been reported to have occurred multiple times between bacteria and nematodes in terrestrial systems [35]. A similar association between a *MutS7*-containing bacterium and an octocoral may have been responsible for HGT of *mtMutS*. Epsilonproteobacteria are common constituents of deep-sea environments [50,51], which also contain diverse octocoral communities (e.g.[52]), therefore associations between the two are possible. The fact that such an occurrence has not yet been documented may simply reflect the lack of study of non-pathogenic bacterial associations in octocorals.

### The functionality and function of mtMutS

Evolutionary theory predicts that a successful HGT integration requires that the transferred gene be active in the new host [34]. Our results have demonstrated for the first time that the octocoral *mtMutS *gene contains all coding necessary for a functional MutS protein. The presence of conserved residues for mismatch recognition in Domain I and ATPase-specific residues in Domain V provide primary evidence for functionality (Figure 3). The presence and conserved order of three MutS protein domains provides secondary evidence (Figure 4) and the conserved conformation model (Figure 5) provides tertiary evidence that mtMutS has the potential to play an active role in the octocoral proteome. Finally, our transcriptional analysis indicates that *mtMutS *is transcribed and available as mRNA for translation (Figure 6).

Successful integration of a horizontally transferred gene into a host genome also requires longevity over evolutionary time [34]. Phylogenetic analyses clearly reveal that the *mtMutS *gene has persisted in octocorals since at least the last common ancestor of all extant species (Figure 2; see also [17]). Given the reduction of gene content in the octocoral mitochondrial genome [2,5] and the frequency of its reorganization through intramitochondrial recombination events [11], it can be deduced that there is strong selection for the continued presence of *mtMutS*. Interestingly, no octocoral has been found to lack *mtMutS *even though the very existence of the hexacoral sister group demonstrates that anthozoans are capable of persisting over geologic time spans without it. Apparently the presence of *mtMutS *provides a selective advantage in octocorals, but one that is unnecessary for normal anthozoan mitochondrial function.

The subcellular target of the *mtMutS *mRNA, and the role of the gene product, remains unknown. The predicted protein structure, including the presence of a mismatch recognition site in Domain I (Figure 3), suggests a role in DNA mismatch repair (MMR) is highly likely. Our findings indicate a reduced rate of mtDNA evolution in at least one gene in the Octocorallia, relative to its sister group, that does not appear to extend to the nuclear genome (Figure 7). The eukaryotic *MSH1 *gene, for example, is a nuclear-encoded gene with a protein product that is translocated to the mitochondrion, where it performs MMR [27,53,54]. The MSH1 protein is known to form homodimers during mismatch binding in the mitochondrion [55], but its interaction with other proteins, particularly an endonuclease for strand excision, is unknown. The dimerization of mtMutS has not been examined, but it is possible that the presence of an HNH endonuclease domain could confer self-sufficiency to the protein [31]. The domain is of a type II endonuclease, which is capable of nicking a single target strand such as the erroneous daughter strand in a mismatched DNA heteroduplex. Although prokaryotic MutS1 and eukaryotic MSH2, 3 and 6 MMR systems require one or more other proteins for excision (MutL+MutH and MLH, respectively) [56], the HNH domain on the C-terminal end of mtMutS could replace their actions, obviating the need for additional MMR components. This self-sufficiency could lend itself to mitochondrial MMR activity since MLH proteins associated with nuclear MMR display no active role in mitochondrial MMR [53,56] and thus may be incapable of translocating across the organellar bilayer.

### Implications and future directions

Regardless of the vector of *mtMutS *HGT, and of specific function, the gene's presence within the mitochondrial genome of octocorals is remarkable in itself. It adds another example of a metazoan subjected to HGT to a list that already includes nematodes [35] and molluscs [57]. Unlike these other cases, however, *mtMutS *represents the only recorded instance of HGT into a metazoan mitochondrion. This necessitates a reconsideration of the mechanisms by which mitochondrial genomes evolve since it allows for the possibility of the introduction of foreign genetic material into a system previously thought to be constrained to inheritance through (typically maternal-) descent. It is also noteworthy that the evolution of *mtMutS *itself seems unconstrained by the reduced rate of mutation seen in other octocoral mitochondrial genes (Figure 7). We have now initiated studies to isolate nuclear *MSH *genes from octocorals and compare their ancestral patterns of diversification to *mtMutS *to determine if this pattern is shared by other MMR genes.

## Conclusions

We have provided consolidating evidence for a non-eukaryotic origin of *mtMutS *and have demonstrated the monophyly of this gene among octocorals and other members of the *MutS7 *lineage. It now seems unequivocal that *mtMutS *arose once during octocorallian evolution, in the common ancestor of all extant lineages, through a horizontal gene transfer event from either an epsilonproteobacterium or a nucleocytoplasmic large DNA virus. This transfer represents the only instance recorded to date of HGT into a metazoan mitochondrion. The transferred gene appears to have been successfully integrated into the host genome, as evidenced by both its persistence over evolutionary time and its transcriptional activity. The mtMutS protein contains conserved amino acid positions and domains necessary for post-replicative DNA mismatch repair, as well as a unique additional domain that may enable it to function in a self-contained manner. Although *mtMutS *function remains unknown, we have demonstrated that at least one mitochondrial gene exhibits significantly lower rates of evolution in octocorals compared to hexacorals, providing evidence that mtMutS-mediated MMR may be operating within the octocoral mitochondrial genome.

## Methods

### MtMutS protein structure & gene tree prediction

All complete *MutS *derived amino acid sequences from octocorals (n = 30) were obtained from GenBank http://www.ncbi.nlm.nih.gov/. These sequences were then used in PSI-BLAST searches to obtain the most similar protein matches from non-octocorallian taxa. In addition, representatives of eubacterial *MutS *and the six eukaryotic *MSH *families were also downloaded for comparison. Eubacterial *MutS *sequences included *Escherichia coli *and bacterial hits to octocoral BLAST searches. All eukaryotic *MSH *sequences were obtained from *Homo sapiens*, *Saccharomyces cerevisiae *and *Nematostella vectensis *genomic datasets (Additional file 1).

The presence and identity of protein family domains ('pfam') within octocoral MutS sequences was inferred from queries in the Simple Modular Architecture Resource Tool (SMART) database of EMBL http://smart.embl-heidelberg.de/. The secondary and tertiary structures of protein sequences were inferred through submission to the I-TASSER server http://zhanglab.ccmb.med.umich.edu/I-TASSER/, which predicts structure and function through comparison to databases of known protein function. The results were compared to predicted structures of the most-similar non-octocorallian BLAST results as well as to the known structure of *E. coli *MutS, as determined by Lamers et al. [36]. In particular, conserved functional residues in each domain were compared for their location within predicted domains and tertiary structures.

All amino acid sequences were aligned with the E-INS-I method in MAFFT [58], using the BLOSUM45 scoring matrix. The output was manually adjusted in MacClade 4.08 (Sinauer Assoc.) using the SMART pfam results to ensure homologous domains were correctly matched in the final alignment. Conserved functional residues or motifs of residues in each domain were also used to assist in the manual adjustment, which produced an alignment of 101 taxa and 1819 characters (available on request from the authors). Trimming the amino acid alignment of ambiguously aligned regions resulted in a truncated alignment of 592 characters, with most trimmed regions in Domain I and the interdomain region between Domain I and Domain III. Excluded regions typically contained gaps for all but a minority of taxa, and were thus unlikely to be informative in the phylogenetic analysis. Importantly, the C-terminal HNH domain was trimmed from the final alignment and excluded from subsequent phylogenetic analyses since a homologous domain was not found among the included eubacterial *MutS *and eukaryotic *MSH *sequences.

MEGA 5.05 [59] was used to calculate maximum parsimony trees and to determine an initial distance correction model for Bayesian phylogenetic analysis. Tree searching used the Close-Neighbor-Interchange method with 1000 initial random trees and a MP search level of three. Clade support within the parsimony tree was calculated using 1000 replicates of nonparametric bootstrapping. The Bayesian analysis was implemented in MrBayes 3.1.2 [60] using four independent MCMC runs, each containing six chains of 10^7 ^generations, with two swaps per generation ('*nswaps *= 2') and trees sampled every 1000 generations. The amino acid distance correction model was adjusted using the '*aamodelpr = mixed*' prior in preliminary runs. Burn-in was determined using Tracer 1.5 http://beast.bio.ed.ac.uk/Tracer and the remaining trees were used to construct a 50% majority-rule consensus tree (the '*allcompat*' option of '*sumt*'). Gene trees obtained by maximum parsimony and Bayesian inference were compared for clade structure and for the determination of the sister taxon to the octocoral *mtMutS *lineage.

### Expression of octocoral *mtMutS*

Gene expression of *mtMutS *in octocorals was tested through comparative amplification and sequencing of genomic DNA and reverse-transcribed mRNA. Fresh tissue samples of *Isis hippuris*, *Nephthya *sp. and *Sarcophyton ehrenbergi *were collected from Heron Island Reef, Australia (23.44710°S, 151.91800°E) and were immediately preserved in both 96% ethanol (for DNA extraction) and RNAlater (Sigma-Aldrich Ltd.) and frozen at -80°C (for RNA extraction). Total genomic DNA extraction used a standard 2XCTAB protocol [61] with a single chloroform extraction. Extraction of total RNA was performed with Trizol (Sigma-Aldrich Ltd.), following the manufacturer's recommended protocol for high-salt precipitation. Total RNA was then treated with 1 μl DNase I (Invitrogen Corp.) prior to reverse-transcription using SuperScriptIII (Invitrogen Corp.) to generate cDNA. Reverse transcriptase-free negative controls were included to check for genomic contamination of total RNA extracts.

Octocoral *mtMutS *in gDNA and cDNA was amplified using 0.22 mM of dNTPs, 3 units of *Taq *polymerase, 1X *Taq *Buffer (50 mM KCl, 10 mM Tris-HCl pH 9.0, 1.5 mM MgCl_2_), 2.9 mM of MgCl_2_, 0.16 μM of each primer and either 1 μl (cDNA) or 2 μl (gDNA) of template DNA. The forward primer (mtMutS93_Fwd: AGTTCTATGAACTTTGGCATGAG) was designed by the authors to anneal near the 5'-end (Domain I) of the octocoral *mtMutS *gene and was paired with an existing reverse primer (Mut3458R: TSGAGCAAAAGCCACTCC) [16]. Although both primers were designed for specificity to *mtMutS*, BLAST searches were conducted on each to ensure nuclear *MSH *or other heterologous loci would not be amplified. Reactions were performed under thermocycler conditions of 94°C for 2 min followed by 35 cycles of 94°C for 60 s, 58°C for 60 s and 72°C for 60 s, with a final extension of 5 minutes at 72°C. Template-free negative PCR controls were included in all reactions to ensure observed products were of octocoral origin.

The size of amplified products from gDNA and cDNA were compared on a 1% agarose gel and RT-PCR controls were included to ensure cDNA amplicons were of mRNA origin. Bands of expected size were excised from the gel and dissolved in 4 volumes of 6 M NaI at 55°C for 5 minutes, followed by the addition of 15 μl hydrated silica suspension. The solution was incubated for a further 10 minutes, spun at maximum speed for 30 s and the supernatant was discarded. Pellets were resuspended in 500 μl of buffer (50 mM NaCl, 10 mM Tris-HCl pH 7.5, 2.5 mM EDTA, 50% ethanol), incubated at 55°C for 5 min, spun down and the supernatant was discarded again. The pellets were washed a second time and the pellets were dried prior to resuspension in 20 μl water.

Direct cycle sequencing of these gel-purified PCR products was performed in both forward and reverse directions in 10 μl volumes using 18-30 ng of template DNA, 8 nmol of primer, 1 μl of BigDye Terminator Ready Reaction Mix (Applied Biosystems) and 1.5 μl of buffer. Cycling was performed at 96°C for 2 minutes, followed by 30 cycles of 96°C for 10 s, 50°C for 5 s and 60°C for 4 minutes. Products were cleaned, precipitated and submitted for capillary sequencing at the Australian Genome Research Facility following their guidelines http://www.agrf.org.au/.

Sequences for each colony were assembled and edited in CodonCode Aligner 3.7.1.1 (CodonCode Corp.) and were submitted for BLASTn searches to ensure they were octocoral in origin and not due to contamination. Corresponding *mtMutS *sequences of gDNA and cDNA for each amplicon were then compared for identity and were deposited into GenBank as accession numbers JN383337-JN383342.

### Comparison of Mitochondrial and Nuclear Rates of Evolution Among Anthozoans

All existing octocoral *mtMutS *and anthozoan cytochrome oxidase subunit I (*COI*) and small ribosomal subunit (18S) DNA sequences over 200 bp in length were obtained from GenBank. The sets of sequences for *mtMutS *and *COI *were aligned according to their amino acid translation frame in MacClade and all three datasets were trimmed to exclude intergenic regions, introns (present in hexacoral *COI*, including actiniarians [AF000023], scleractinians [FJ345452, FJ345449] and antipatharians [FJ597643]) and any ambiguous sections of alignment. Incomplete 5' and 3' ends were coded as missing data ("?") for sequences not spanning the length of the genes. Redundant sets of sequences were each collapsed into a single, multi-species taxon and the resulting matrix was used to calculate uncorrected pairwise distances among octocoral *mtMutS *sequences, octocoral *COI *sequences, hexacoral *COI *sequences, octocoral 18S sequences and hexacoral 18S sequences in MEGA. Mann-Whitney *U*-tests were used to test for significant differences in the mean pairwise differences between octocoral *mtMutS *and octocoral *COI*, between octocoral *COI *and hexacoral *COI *and between octocoral 18S and hexacoral 18S.

## Authors' contributions

JB and SD together conceived and designed the study. JB conducted the phylogenetic, gene structure, protein structure and gene expression analyses, and drafted the manuscript. SD advised on data analyses and helped to draft the manuscript. Both authors read and approved the final manuscript.

## Supplementary Material

Additional file 1 **List of GenBank sequences used in phylogenetic reconstructions and homology assessments**. A list of GenBank sequences used to reconstruct the phylogenies of *mtMutS/MutS/MSH *and the *mtMutS *protein domains and structural conformation. 'Phylogenetic Position' refers to the clade in Figure 2 that was occupied by each sequence. File provided in pdf format. Click here for file
