# Supplementary material for: A unique horizontal gene transfer event has provided the octocoral mitochondrial genome with an active mismatch repair gene that has potential for an unusual self-contained function
Source: BMC Evol Biol. 2011 Jul 29;11:228. doi: 10.1186/1471-2148-11-228 (PMC3166940; doi:10.1186/1471-2148-11-228)
Supplement: Additional file 1 — List of GenBank sequences used in phylogenetic reconstructions and homology assessments. A list of GenBank sequences used to reconstruct the phylogenies of mtMutS/MutS/MSH and the mtMutS protein domains and structural conformation. 'Phylogenetic Position' refers to the clade in Figure 2 that was occupied by each sequence. File provided in pdf format. [file 1471-2148-11-228-S1.PDF]

Phylogenetic Position	Taxon Name	GenBank I.D.
OCTOCORAL *mtMutS* CLADE		
	Acanella eburnea	YP_001994427
	Acanthogorgia sp	AAP92778
	Briareum asbestinum	ABF69703
	Calyptrophora sp	ACB28528
	Chrysogorgia sp 1	ADD13036
	Chrysogorgia sp 2	ADJ53944
	Chrysogorgia sp 3	ADJ53948
	Chrysogorgia sp 4	ADJ53950
	Chrysogorgia sp 5	ADJ53952
	Chrysogorgiidae	ADJ53919
	Corallium ducale	ACB28532
	Dendronephthya gigantea	YP_003331255
	Iridogorgia sp	ADJ53940
	Iridogorgia fontinalis	ACB28529
	Keratoisidinae	ABQ86201
	Lepidisis sp	ADJ95230
	Leptogorgia chilensis	AAP92777
	Leptogorgia hebes	AAP92776
	Leptogorgia virgulata	AAP92775
	Metallogorgia melanotrichos	ADJ53936
	Narella dichotoma	ACB28527
	Paramuricea sp	ACB28526
	Protoptilum sp	ACB28531
	Pseudopterogorgia bipinnata	ABF93177
	Radicipes	ADD13105
	Radicipes sp 1	ADJ53946
	Radicipes sp 2	ADJ53942
	Renilla reniformis	ACB28530
	Rhodaniridogorgia sp	ADJ53938
	Sarcophyton glaucum	O63852
EUKARYOTA *MSH* CLADE		
	Homo sapiens (MSH2)	NP_000242
	Homo sapiens (MSH3)	NP_002430
	Homo sapiens (MSH4)	AAB72039
	Homo sapiens (MSH5)	BAB63375
	Homo sapiens (MSH6)	NP_000170
	Saccharomyces cerevisiae (MSH2)	NP_014551
	Saccharomyces cerevisiae (MSH3)	NP_010016
	Saccharomyces cerevisiae (MSH4)	NP_116652
	Saccharomyces cerevisiae (MSH5)	NP_010127
	Saccharomyces cerevisiae (MSH6)	NP_010382
	Nematostella vectensis (MSH2)	XP_001630382
	Nematostella vectensis (MSH3)	XP_001630862
	Nematostella vectensis (MSH4)	XP_001627391
	Nematostella vectensis (MSH5)	XP_001640822
NCLDV *MutS* CLADE		
	Acanthamoeba polyphaga mimivirus	YP_142713
	Cafeteria roenbergensis virus	YP_003970119
	Chrysochromulina ericina virus	CBX20920
	Heterocapsa circularisquama virus	BAJ49801
	Organic Lake phycodnavirus	ADX06024
	Phaeocystis pouchetii virus	CBX20917
	Pyramimonas orientalis virus	CBX20916

Phylogenetic Position	Taxon Name	GenBank I.D.
EUBACTERIA *MutS* & EUKARYOTA *MSH1* CLADE		
	Saccharomyces cerevisiae (MSH1)	NP_011988
	Nematostella vectensis (MSH1)	XP_001618609
	Escherichia coli	CAQ33065
	Marivirga tractuosa	YP_004054386
	Kordia algicida	ZP_02160260
	Gramella forsetii	YP_860313
	Polaribacter irgensii	ZP_01117326
	Flavobacteria bacterium	ZP_01201039
	Psychroflexus torquis	ZP_01252139
	Thermotoga naphthophila	YP_003346588
	Dictyoglomus thermophilum	YP_002250799
	Dictyoglomus turgidum	YP_002352970
	Megamonas hypermegale	CBL07203
	Clostridium acetobutylicum	NP_348461
	Thermotoga petrophila	YP_001244609
	Alkaliphilus metalliredigens	YP_001320356
	Thermotoga sp	YP_001739144
	Natranaerobius thermophilus	YP_001917667
	Candidatus Amoebophilus asiaticus	YP_001957876
	Thermodesulfovibrio yellowstonii	YP_002248548
	Staphylococcus lugdunensis	YP_003471814
	Croceibacter atlanticus	YP_003715795
	Acetohalobium arabaticum	YP_003827743
	Butyrivibrio proteoclasticus	YP_003830810
	Ruminococcus gnavus	ZP_02041118
	Anaerococcus hydrogenalis	ZP_03304309
	Anaerococcus lactolyticus	ZP_03915301
	Shuttleworthia satelles	ZP_04455126
	Lactobacillus jensenii	ZP_04645907
	Bryantella formatexigens	ZP_05345491
	Anaerococcus vaginalis	ZP_05472524
	Lactobacillus coleohominis	ZP_05553412
	Lactobacillus jensenii	ZP_05866182
	Selenomonas sputigena	ZP_05898570
	Prevotella sp	ZP_60406096
	Lactobacillus plantarum	ZP_07079609
	Chryseobacterium gleum	ZP_07084472
	Algoriphagus sp	ZP_07718872
	Lactobacillus oris	ZP_07730580
EPSILONPROTEOBACTERIA *MutS* CLADE		
	Arcobacter butzleri	YP_001489403
	Arcobacter nitrofigilis	ADG92380
	Campylobacterales bacterium	ZP_05072493
	Nautilia profundicola	YP_002607133
	Nitratifractor salsuginis	YP_004167604
	Nitratiruptor sp	YP_001355988
	Sulfurimonas denitrificans	YP_393958
	Sulfurospirillum deleyianum	YP_003303695
	Sulfurimonas autotrophica	YP_003892528
	Sulfuricurvum kujiense	YP_004059760
	Uncultured organism	ADD95762
